# TEE-Guided RIJ Approach to AngioVac Extraction of a Large Pulmonic Vegetation

**DOI:** 10.1016/j.jscai.2022.100430

**Published:** 2022-08-12

**Authors:** Damian Valencia, Naveen Vuppuluri, Ananya Reddy, Niranjan Reddy, Raja Nazir, David Stultz

**Affiliations:** a Department of Cardiovascular Medicine, Kettering Health, Kettering, Ohio; b Boonshoft School of Medicine, Wright State University, Dayton, Ohio; c Department of Internal Medicine, Kettering Health, Kettering, Ohio; d Department of Medical Sciences, University of Cincinnati, Cincinnati, Ohio

**Keywords:** AngioVac, echocardiography, endocarditis, extraction, percutaneous intervention, pulmonic valve vegetation

**Figure 1:**
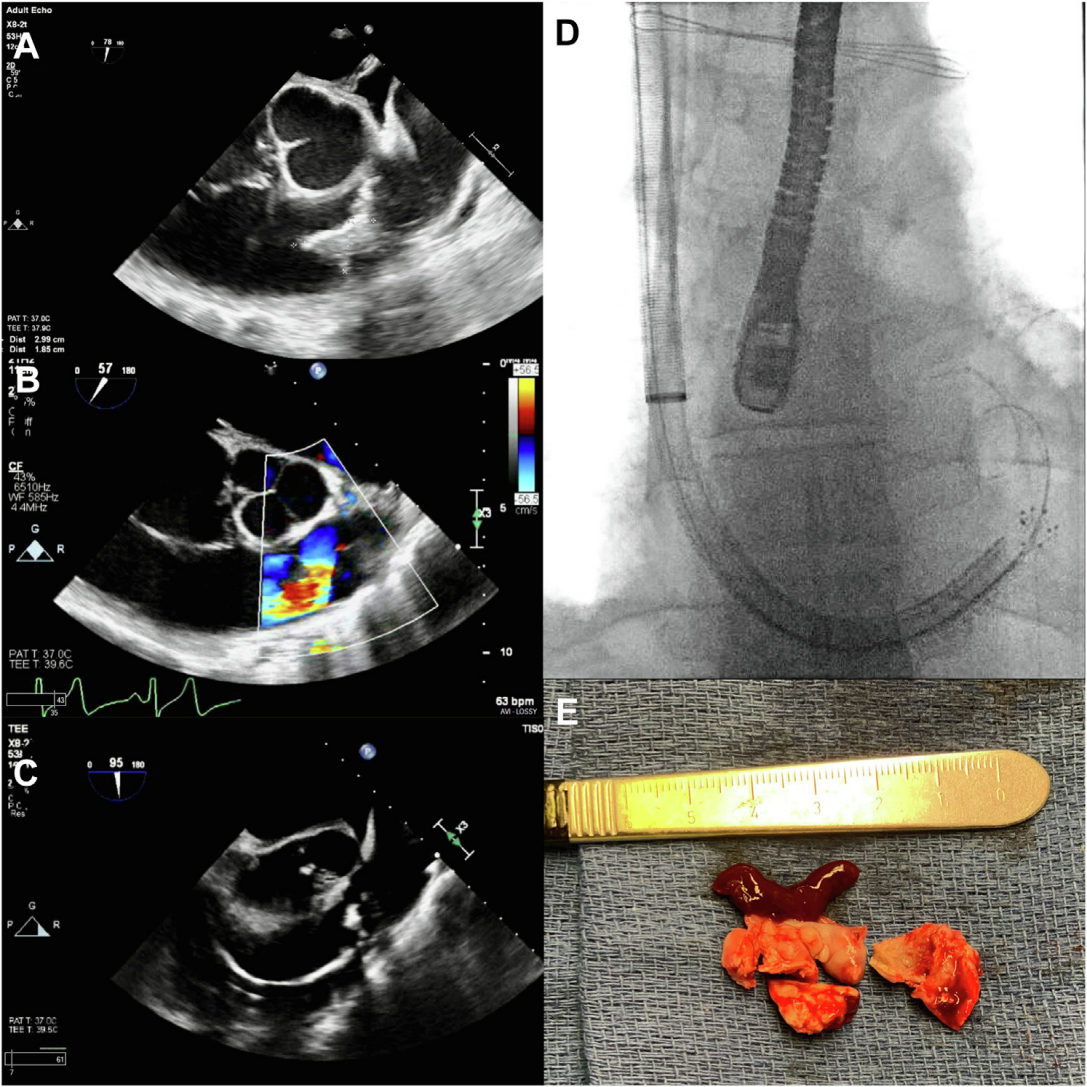
**Transesophageal echocardiography, fluoroscopy, and visual images of the pulmonic valve vegetation.** (**A**) Transesophageal echocardiography in the short-axis midesophageal view at 78° showing a 2.99 × 1.85-cm vegetation on the right cusp of the pulmonic valve during ventricular diastole. (**B**) Transesophageal echocardiography color Doppler in the short-axis midesophageal view at 57° showing severe pulmonic valve regurgitation. (**C**) Transesophageal echocardiography in the short-axis midesophageal view at 95° showing the AngioVac cannula in the right ventricular outflow tract at the level of the pulmonic valve. (**D**) Fluoroscopy confirming the AngioVac cannula in the right ventricular outflow tract at the level of the pulmonic valve. The transesophageal echocardiography probe can also be seen in the midesophageal position. (**E**) The extracted specimen, which appears to be a combination of vegetation and thrombus, can be seen next to a scalpel with centimeter markings.

The AngioVac System (AngioDynamics, Inc) is currently approved for the removal of right-sided venous thrombi and emboli.^1^ This case highlights its off-label use in the extraction of large pulmonic valve vegetation. The patient was a chronically ill 45-year-old man with a past medical history of chronic systolic heart failure with reduced ejection fraction (left ventricular ejection fraction, 30%-35%) from nonischemic cardiomyopathy, chronic hypoxic respiratory failure (2 L nasal cannula oxygen at baseline), chronic kidney disease (stage IIIb), severe pulmonary hypertension (mean resting pulmonary artery pressure, 60 mm Hg), recurrent episodes of bacteremia with right-sided infective endocarditis from intravenous drug use, and medical noncompliance. The patient was admitted to our facility for septic shock with bacteremia (methicillin-resistant *Staphylococcus aureus* and *Bacteroides fragilis* positive cultures), acute hypoxic respiratory failure with bilateral pneumonia (believed to be from septic emboli), and acute systolic heart failure exacerbation with presumed recurrent infective endocarditis. The patient was intubated and on multiple high dose vasopressors (norepinephrine bitartrate, vasopressin, and epinephrine) and intravenous antibiotics per culture sensitivities (daptomycin and linezolid). The patient was also placed on continuous renal replacement therapy because of anuric acute renal failure. Because of poor transthoracic echocardiographic windows and image quality, a transesophageal echocardiogram (TEE) was pursued. The TEE showed a large mobile pulmonic valve vegetation (Figure 1A) measuring 2.99 × 1.85 cm, associated with severe regurgitation (Figure 1B). Because of persistently positive blood cultures after 5 days of antibiotics, cardiothoracic surgery and interventional cardiology departments were consulted for vegetation extraction. Surgical intervention was deemed of high-to-prohibitive risk because of the severity of acute illness described above; the decision was made for a percutaneous approach utilizing the AngioVac System. The patient was taken to the operating room, and a TEE probe was inserted and maintained in the midesophageal position. Ultrasound-guided access of the right internal jugular vein and right common femoral vein was obtained using the Seldinger technique. A 26-F Gore dry seal sheath was placed in the right internal jugular vein (AngioVac cannula site), and an 18-F cannula was placed in the right common femoral vein (reinfusion site) after appropriate successive dilations. After anatomical considerations, the right internal jugular vein was chosen as the AngioVac cannula site to allow for easier placement in the right ventricular outflow tract. Weight-based heparin was administered for a goal activated clotting time of >250 seconds. A 0.035-inch double curve Lunderquist extra-stiff wire guide (Cook Medical) was advanced and positioned in the right interlobar pulmonary artery. A second-generation 20**°** angled AngioVac System cannula was then positioned in the right ventricular outflow tract using the over-the-wire technique, and aspiration was performed. The cannula was then advanced up to the pulmonic valve under TEE and fluoroscopic guidance (Figure 1C, D) for vegetation debulking. The aspiration time was 4 minutes and 18 seconds. Five separate vegetative lesions were aspirated, one of which appeared to be mostly thrombus. The filtered blood was returned through the right femoral cannula. The device was removed, and anticoagulation was reversed using protamine. The right internal jugular and right common femoral veins were closed with the Perclose ProGlide Suture-Mediated Closure System (Abott). The extracted specimen appeared to be a combination of vegetation and thrombus (Figure 1E). The final combined measurement of the lesion was 3.5 × 3.0 × 0.5 cm. The specimen was sectioned and examined under a microscope, revealing organizing fibrin and calcifications consistent with the diagnosis of pulmonic valve vegetation. Postoperative TEE imaging did not show any evidence of residual vegetation, only mild thickening of the pulmonic valve. The pulmonic valve did not appear to be damaged, with only a single mild regurgitation jet at the coaptation line. There were no immediate postoperative complications noted. Repeat blood cultures after vegetation extraction were negative. The patient was weaned from the ventilator and extubated as hemodynamics stabilized. The patient was discharged to a rehabilitation facility on a 6-week course of intravenous antibiotics. Although the AngioVac device has been designed for intravascular and pulmonary thrombectomy, it can be utilized in select cases for right-sided vegetation extraction when surgical risk is high or prohibitive, as illustrated in this case.
